# Benefits and risks of clofarabine in adult acute lymphoblastic leukemia investigated in depth by multi‐state modeling

**DOI:** 10.1002/cam4.6756

**Published:** 2024-04-29

**Authors:** Sjoerd J. F. Hermans, Yvette van Norden, Jurjen Versluis, Anita W. Rijneveld, Bronno van der Holt, Okke de Weerdt, Bart J. Biemond, Arjan A. van de Loosdrecht, Lotte E. van der Wagen, Mar Bellido, Michel van Gelder, Walter J. F. M. van der Velden, Dominik Selleslag, Daniëlle van Lammeren‐Venema, Vincent H. J. van der Velden, Liesbeth C. de Wreede, Douwe Postmus, Francesco Pignatti, Jan J. Cornelissen

**Affiliations:** ^1^ Erasmus University Medical Center Cancer Institute Rotterdam The Netherlands; ^2^ HOVON Foundation Rotterdam The Netherlands; ^3^ Department of Hematology Sint Antonius Hospital Nieuwegein The Netherlands; ^4^ Department of Hematology Amsterdam University Medical Centers, Amsterdam Medical Center Amsterdam The Netherlands; ^5^ Department of Hematology Cancer Center Amsterdam, Amsterdam University Medical Centers, Vrije Universiteit University Medical Center Amsterdam The Netherlands; ^6^ Department of Hematology University Medical Center Utrecht The Netherlands; ^7^ Department of Hematology University Medical Center Groningen Groningen The Netherlands; ^8^ Department of Hematology Maastricht University Medical Center Maastricht The Netherlands; ^9^ Department of Hematology Radboud University Medical Center Nijmegen The Netherlands; ^10^ St Jan Hospital Bruges Belgium; ^11^ Department of Hematology Haga Teaching Hospital The Hague The Netherlands; ^12^ Department of Immunology Erasmus MC, University Medical Center Rotterdam Rotterdam The Netherlands; ^13^ Department of Biomedical Data Sciences Leiden University Medical Center Leiden The Netherlands; ^14^ Department of Epidemiology University of Groningen, University Medical Center Groningen Groningen The Netherlands; ^15^ Oncology and Hematology Office European Medicines Agency Amsterdam The Netherlands

**Keywords:** acute lymphoblastic leukemia, clofarabine, MRD, multi‐state modeling, off‐protocol treatment, transition probability

## Abstract

**Background:**

We recently reported results of the prospective, open‐label HOVON‐100 trial in 334 adult patients with acute lymphoblastic leukemia (ALL) randomized to first‐line treatment with or without clofarabine (CLO). No improvement of event‐free survival (EFS) was observed, while a higher proportion of patients receiving CLO obtained minimal residual disease (MRD) negativity.

**Aim:**

In order to investigate the effects of CLO in more depth, two multi‐state models were developed to identify why CLO did not show a long‐term survival benefit despite more MRD‐negativity.

**Methods:**

The first model evaluated the effect of CLO on going off‐protocol (not due to refractory disease/relapse, completion or death) as a proxy of severe treatment‐related toxicity, while the second model evaluated the effect of CLO on obtaining MRD negativity. The subsequent impact of these intermediate events on death or relapsed/refractory disease was assessed in both models.

**Results:**

Overall, patients receiving CLO went off‐protocol more frequently than control patients (35/168 [21%] vs. 18/166 [11%], *p* = 0.019; HR 2.00 [1.13–3.52], *p* = 0.02), especially during maintenance (13/44 [30%] vs. 6/56 [11%]; HR 2.85 [95%CI 1.08–7.50], *p* = 0.035). Going off‐protocol was, however, not associated with more relapse or death. Patients in the CLO arm showed a trend towards an increased rate of MRD‐negativity compared with control patients (HR MRD‐negativity: 1.35 [0.95–1.91], *p* = 0.10), which did not translate into a significant survival benefit.

**Conclusion:**

We conclude that the intermediate states, i.e., going off‐protocol and MRD‐negativity, were affected by adding CLO, but these transitions were not associated with subsequent survival estimates, suggesting relatively modest antileukemic activity in ALL.

## Graphical abstract

We developed two multi‐state models to identify in more depth why clofarabine (CLO) did not show a long‐term survival benefit despite more MRD‐negativity, as recently reported in the HOVON‐100 trial. Patients receiving CLO went off‐protocol (indicated by the left figures) more frequently than control patients (HR 2.00 [1.13–3.52], *p* = 0.02)—especially during maintenance (HR 2.85 [95%CI 1.08–7.50], *p* = 0.035)—and showed a trend towards an increased rate of MRD‐negativity (indicated by the right figures) (HR 1.35 [0.95–1.91], *p* = 0.10); however, neither transition significantly affected subsequent survival estimates. We conclude that the intermediate states, i.e., going off‐protocol and MRD‐negativity, were affected by adding CLO, but these transitions were not associated with subsequent survival estimates, suggesting relatively modest antileukemic activity in ALL.

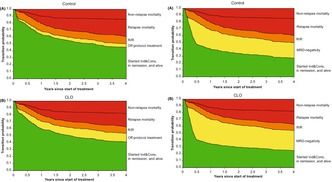

## 1 INTRODUCTION

Current drug development in upfront therapy for adult patients with acute lymphoblastic leukemia (ALL) focuses on prevention of relapse, as most newly diagnosed adult ALL patients may achieve complete remission (CR) upon intensive induction therapy. Clofarabine (CLO) did not improve event‐free survival (EFS) when added to induction/consolidation therapy in adult ALL, as recently reported.^1^ While CLO increased the proportion of measurable residual disease (MRD)‐negative patients by 20%,^1^ it did not reduce the relapse rate and did not improve long‐term survival outcomes. Meanwhile, patients treated with CLO experienced more treatment‐related toxicity, as measured by adverse events ≥grade 3. Treatment‐related toxicity was proposed as a possible cause for the lack of long‐term benefit. However, the impact of toxicities on subsequent efficacy endpoints could not be reliably assessed by conventional Cox‐regression. We therefore applied multi‐state modeling to evaluate the impact of intermediate events, including severe toxicities and MRD‐negativity, on long‐term efficacy estimates.

Multi‐state modeling, developed by Philip Hougaard^2^ and Per Kragh Andersen^3^ amongst others, is a more flexible approach that takes intermediate events into account and allows for estimation of probabilities of both intermediate events and endpoints. Distinct treatment phases may be modeled as separate, intermediate events, which allows to precisely identify which patients transition to what event at which time point. Previously, multi‐state modeling was applied by our group to further elucidate the efficacy of CLO in acute myeloid leukemia (AML) as reported in the randomized phase III HOVON102/SAKK30/09 trial. The multi‐state re‐analysis by Bakunina et al. identified that addition of CLO, independent of MRD status or allogeneic stem cell transplant, was associated with a reduction of relapse, but also translated into increased treatment‐related mortality.^4^, ^5^ Based on these observations in AML and the finding that CLO did not improve EFS in ALL, we hypothesized that addition of CLO to first‐line intensive treatment for ALL was also associated with an increase in treatment‐related mortality at the cost of an improved antileukemic effect (i.e., reduction of relapse). To investigate this hypothesis in depth, we analyzed the HOVON‐100 ALL trial in an alternative way using intermediate events to more specifically address the impact of CLO on intermediate events and outcome estimates at any time‐point during trial treatment. We constructed two separate multi‐state models, one specific to severe toxicity (i.e., going off‐protocol), and one specific to efficacy (i.e., MRD negativity). As such, we aimed to further clarify the potential treatment benefits and risks of CLO.

## 2 PATIENTS AND METHODS

### 2.1 HOVON‐100 trial characteristics and outcomes

The HOVON‐100 phase III clinical trial evaluated the efficacy and safety of CLO added to standard intensive therapy in newly diagnosed adult ALL patients aged between 18 and 70 years.^1^ CLO was added to the prephase and consolidation phase of a pediatric‐inspired treatment protocol, of which the intensity of the chemotherapy backbone was age‐dependent. The efficacy of CLO was determined by EFS (primary endpoint), and overall survival (OS) (secondary endpoint), while safety was evaluated by the occurrence of adverse events, and stopping of protocol treatment for other reasons than completion, relapsed or refractory ALL, or death. Follow‐up data were available until February 1, 2023.

A total of 166 and 168 patients were randomized to the control and CLO arm, respectively. Overall, patients had a median age of 43 years (range: 18–70), which was comparable between treatment arms (Table 1). Patients were male in 58% and 60% in the control and CLO arm, respectively. Most patients were classified as “high risk” according to ALL risk classification based on white blood cell count, no complete hematological remission (CR) after the first remission‐induction course and cytogenetic or molecular aberrations. Patients in both treatment arms achieved a CR on protocol in 89% of cases. The cumulative incidence of relapse at 5 years was 23 ± 3% and 17 ± 3% in the control and CLO arm, respectively. Median follow‐up for all patients was 7.0 years as estimated with inverse Kaplan–Meier analysis. Five‐year EFS was 50 ± 4% for patients in the control arm, and 53 ± 4% for patients in the CLO arm. OS at 5 years was estimated at 61 ± 4% for patients in both study arms.

**TABLE 1:** HOVON‐100 ALL trial: patient characteristics and outcomes.

Parameter	Control	CLO
No. of patients	166	168
Age, median (range)	42 (18–70)	43 (18–70)
Sex, *n* (%)		
Male	96 (57.8)	100 (59.5)
Female	70 (42.2)	68 (40.5)
ALL risk classification		
Standard	64 (38.6)	60 (35.7)
High	102 (61.4)	108 (64.3)
Complete remission on protocol		
Yes	148 (89.2)	149 (88.7)
No	18 (10.8)	19 (11.3)
MRD during consolidation treatment		
Positive	22 (13.3)	6 (3.6)
Negative	54 (32.5)	75 (44.6)
Missing	90 (54.2)	87 (51.8)
Maintenance treatment*, *n* (%)	57 (34.3)	44 (26.2)
alloSCT*, *n* (%)	70 (42.2)	70 (41.7)
Off‐protocol treatment, *n* (%)	18 (10.8)	35 (20.8)
5‐year cumulative incidence of relapse (estimate ± SE)	23 ± 3	17 ± 3
5‐year cumulative incidence of NRM (estimate ± SE)	11 ± 2	16 ± 3
5‐year EFS (estimate ± SE)	50 ± 4	53 ± 4
5‐year OS (estimate ± SE)	61 ± 4	61 ± 4

*Note*: Number and percentage for maintenance treatment, alloSCT, and off‐protocol treatment are based on raw numbers and do not take censoring into account.

Abbreviations: ALL, acute lymphoblastic leukemia; alloSCT, allogeneic stem cell transplantation; CLO, clofarabine; MRD, measurable residual disease; NRM, non‐relapse mortality.

* Numbers and percentages are based on on‐protocol administered treatment.

### 2.2 Multi‐state analysis

To identify why CLO did not show a long‐term survival benefit despite more MRD‐negativity, we included all (*n* = 334) patients eligible for analysis in the clinical trial. In two separate multi‐state models, time was measured from the start of induction & consolidation chemotherapy (Ind&Cons) until the date of last contact. Patients remained in the preceding state until an event occurred that caused the patient to enter a subsequent state. This approach allowed for the evaluation of patients during distinct treatment phases, such as maintenance treatment, as compared to evaluating only endpoints.

#### 2.2.1 Model 1: Off‐protocol model

**FIGURE 1:**
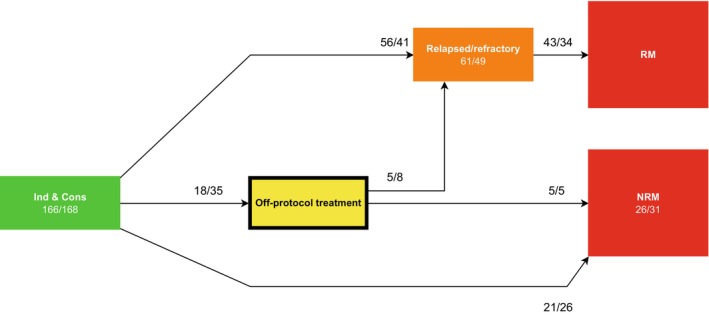
Multi‐state model for off‐protocol treatment. A time‐inhomogeneous Markov model for the events between Induction & Consolidation (Ind&Cons) and date of last contact was developed. All patients started in Ind&Cons at time 0 and could enter a subsequent state only following relapsed/refractory acute lymphoblastic leukemia (ALL), going off‐protocol, or NRM. Patients who did not qualify for a subsequent state remained in the preceding state. Going off‐protocol for reasons other than relapsed/refractory disease, death or treatment completion was used as a proxy of treatment‐related toxicity by CLO. Going off‐protocol, and relapsed/refractory ALL were used as intermediate events, while RM and NRM were modeled as end states. Event counts per treatment arm (“control vs. CLO”) are listed for each transition. NRM, non‐relapse mortality; RM, relapse mortality (all mortality taking place after relapsed/refractory disease).

To evaluate the effect of CLO with respect to treatment‐related toxicity, we developed a Markov time‐inhomogeneous multi‐state model describing the events between Ind&Cons (start state) until the date of last contact. Going off‐protocol, and relapsed/refractory ALL were modeled as intermediate events, while relapse mortality (RM) and non‐relapse mortality (NRM) were considered end states (state entry criteria are defined in Table S1). Following Ind&Cons, patients were at risk for transitioning to an intermediate, or end state, as indicated by the arrows (Figure 1). Treatment‐related toxicity was modeled indirectly using the off‐protocol treatment state as a substitute, allowing patients only to enter into this state if they went off‐protocol for reasons other than relapsed/refractory ALL, death, or completion of protocol treatment. The relative effect of CLO on going off‐protocol, and relapsed/refractory disease was assessed using a transition‐specific Cox model that included treatment arm as covariate with control treatment as reference. We performed a sensitivity analysis in an extended off‐protocol model including on‐protocol allogeneic stem cell transplant (alloSCT) and maintenance treatment as intermediate events to identify during which treatment phase patients receiving CLO had a possibly increased risk of going off‐protocol (Figure S1).

#### 2.2.2 Model 2: MRD model

**FIGURE 2:**
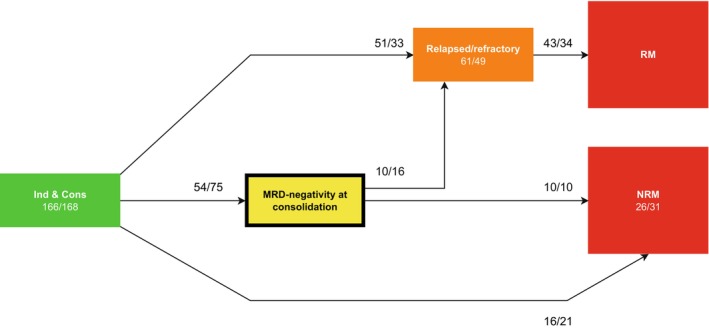
Multi‐state model for MRD. A time‐inhomogeneous Markov model for the events between Induction & Consolidation (Ind&Cons) and date of last contact was developed. All patients started in Ind&Cons at time 0 and could enter a subsequent state only following relapsed/refractory disease, obtaining MRD‐negativity during consolidation treatment, or NRM. Patients who did not qualify for a subsequent state remained in the preceding state. MRD‐negativity was defined as MRD <10^−4^ using flow cytometry or polymerase chain reaction. MRD‐negativity at consolidation, and relapsed/refractory disease were used as intermediate events, while RM and NRM were modeled as end states. Event counts per treatment arm (“control vs. CLO”) are listed for each transition. NRM, non‐relapse mortality; RM, relapse mortality (all mortality taking place after relapsed/refractory disease).

With respect to the potential benefit of CLO, a second Markov time‐inhomogeneous multi‐state model was developed that incorporated MRD‐negativity after consolidation chemotherapy as an intermediate event (Table S1). Going off‐protocol was not considered in this multi‐state model. All other states were evaluated according to the off‐protocol treatment model. A schematic overview of the MRD‐negativity model is shown in Figure 2. MRD‐negativity was defined as no evidence of disease with a flow cytometry or polymerase chain reaction threshold of <10^−4^. MRD at consolidation was available in 157 (47%) patients and was incorporated in the analysis differently compared with the original analysis.^1^ Rijneveld et al.^1^ evaluated MRD (positive vs. negative) at first consolidation only in patients who had achieved CR on protocol and had known MRD outcome. Patients without MRD assessment after consolidation were included in the analysis presented here for developing the multi‐state “MRD” model and were considered MRD positive, irrespective of CR status. Missing MRD data were not imputed. This approach resulted in MRD‐negativity in 54/166 (33%) of control patients and in 75/168 (45%) of patients in the CLO arm (Table 1).

### 2.3 Statistical software

All analyses were performed using R (version 4.2.1 or higher) developed by the R Core Team. The *survival* and *mstate* ^6^, ^7^ packages were used for the time‐to‐event and multi‐state analyses, whereas packages *haven*, *magrittr*, *dplyr*, and *tidyr* were used for data management.

## 3 RESULTS

### 3.1 Model 1: Off‐protocol treatment

**FIGURE 3:**
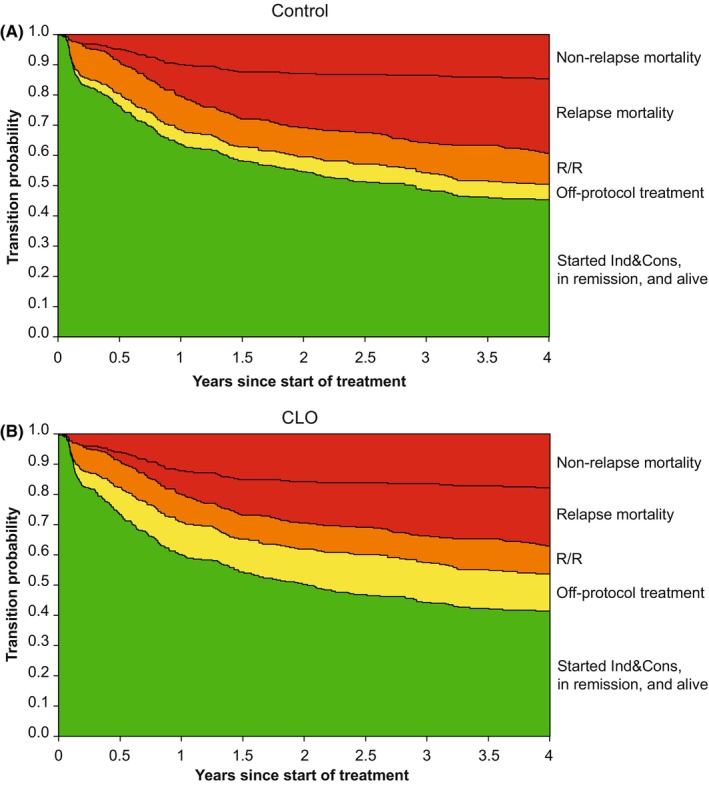
Transition probabilities to all states from Ind&Cons relating to the off‐protocol treatment model (Figure 1). Starting in Ind&Cons at time 0, the distance between neighboring lines depicts the probability of being in that state, at each time point. Probabilities of intermediate states can both increase and decrease over time as patients may enter and leave these states. (A) Transition probabilities for patients allocated to the control arm. (B) Transition probabilities for patients allocated to the CLO arm. Ind&Cons, Induction & Consolidation treatment; R/R, relapsed/refractory disease; Relapse mortality, all mortality taking place after relapsed/refractory disease.

A total of 53 patients went off‐protocol, which was *n* = 18 (11%) in the control arm and *n* = 35 (21%) in the CLO arm (Figure 1; Table 1). Rijneveld et al.^1^ identified 55 patients as going off‐protocol without considering prior on‐protocol treatment. Two of those 55 patients (4%) were not regarded as “off‐protocol” in the analysis presented here, as both patients were treated with an alloSCT on‐protocol. Thus, 53 off‐protocol patients remained (control: *n* = 18; CLO: *n* = 35). Patients proceeded to maintenance treatment or alloSCT in 34% and 42% of cases, respectively, in the control arm, whereas maintenance treatment or alloSCT were applied in 26% and 42% of cases, respectively, in the CLO arm (Table 1). Next, a multi‐state analysis was performed to assess the impact of CLO on going off‐protocol (Figure 1). In total, 334 patients started in the Ind&Cons state. Thirty‐four patients went off protocol during the Ind&Cons phase of protocol treatment and 19 patients during maintenance treatment. Patients receiving CLO went off‐protocol more frequently than control patients (35/168 [21%] vs. 18/166 [11%], *p* = 0.019 [Chi‐squared test]). As shown in Figure 1, 110 patients experienced ALL relapse or refractory disease, of whom 77 died. Control patients proceeded to the relapsed/refractory disease, RM, and NRM states in comparable proportions with CLO patients (Figure 1, Figure S1). Figure 3AB depicts the transition probabilities of the off‐protocol treatment model by treatment arm from the start of Ind&Cons until the end states, namely RM, and NRM. A higher probability of going off‐protocol (depicted in yellow) was apparent in CLO treated patients (Figure 3A vs. 3B; Figure S2; Table S2; Table S7).

**FIGURE 4:**
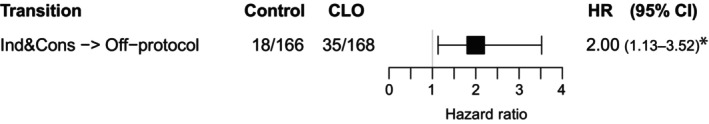
Forest plot for the transition to the off‐protocol treatment state as depicted in Figure 1. The estimate shown is a hazard ratio of a semi‐parametric Cox model including treatment arm (CLO vs. control). A hazard ratio smaller than 1 (left of the vertical line) indicates a lower risk of going off‐protocol for the CLO arm. The number of patients (relative to number entering the respective state) making the transition to the off‐protocol state by treatment arm are tabulated in the middle columns. CI, confidence interval; CLO, number of patients of the CLO arm making the transition; Control, number of patients of the control arm making the transition; HR, hazard ratio; Ind&Cons, Induction & Consolidation treatment. * The transition from Ind&Cons to the off‐protocol treatment state was the only transition with a *p*‐value less than 0.05 (0.02).

Transition‐specific Cox models were used to quantify the effect of CLO versus control treatment on the transition from Ind&Cons to the off‐protocol treatment state (Figure 4). Overall, patients receiving CLO had a significantly increased risk of going off‐protocol (HR: 2.00 [1.13–3.52], *p* = 0.02). Next, we quantified the effect of CLO on going off‐protocol in the extended off‐protocol model. Patients in the CLO arm showed a non‐significant increased risk (HR: 1.68 [0.83–3.40], *p* = 0.15) of going off‐protocol during Ind&Cons, but exhibited a 2.85‐fold increased risk (95%CI: 1.08–7.50, *p* = 0.035) of going off‐protocol during maintenance treatment, compared with control patients (Figure S3). The 53 patients in the off‐protocol treatment state went off‐protocol for different reasons, including 44 patients (83%) due to treatment‐related toxicity, four patients (7.5%) due to treatment delay, three patients (5.7%) based on a revision of the initial diagnosis (e.g., CML blast crisis), and two patients (3.8%) based on revision of treatment response. A total of 80 adverse events (CLO: 59 events; control: 21 events) were observed for these 53 patients (CLO: 35 patients; control: 18 patients) in the 30‐day period before going off‐protocol. These adverse events were related to infections in 34% versus 17% of the patients, to increased liver enzymes in 20% versus 17%, and to gastro‐intestinal toxicity in 9% versus 6%, for CLO versus control, respectively.

HRs (CLO vs. control treatment) corresponding to all transitions in the off‐protocol models are shown in Table S3 and Table S8. The effect of going off‐protocol on relapsed/refractory disease was analyzed by comparing the risk of relapse in patients who went off‐protocol versus those who continued protocol treatment. Ninety‐seven out of 334 (29%) patients transitioned from Ind&Cons to relapsed/refractory disease, whereas 13 out of 53 (25%) patients transitioned from the off‐protocol treatment state to relapsed/refractory disease. Going off‐protocol was not significantly associated with an increased risk of relapsed/refractory disease (HR: 1.54 [0.85–2.80], *p* = 0.16), while adjusting for treatment arm.

### 3.2 Model 2: MRD model

**FIGURE 5:**
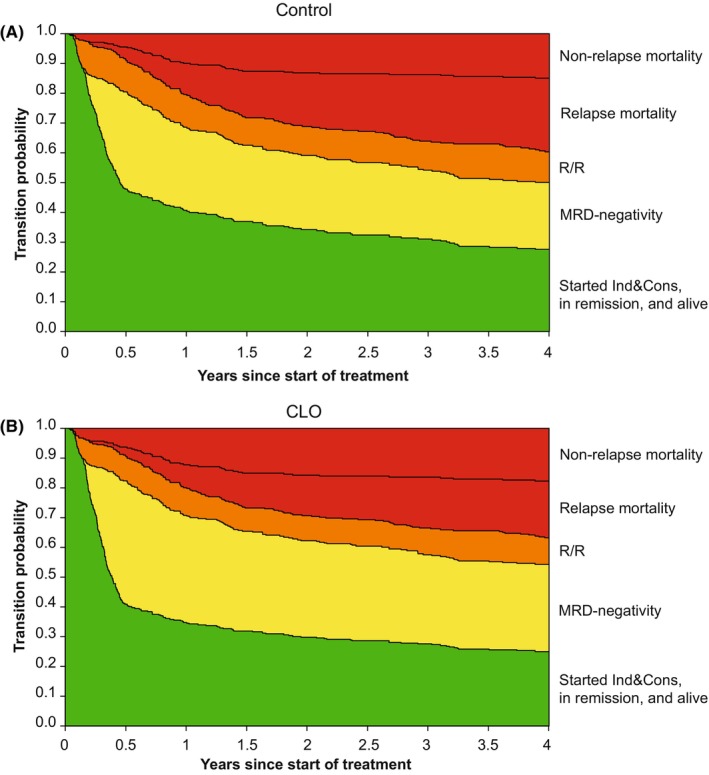
Transition probabilities to all states from Ind&Cons relating to the MRD model (Figure 2). Starting in Ind&Cons at time 0, the distance between neighboring lines depicts the probability of being in that state, at each time point. Probabilities of intermediate states can both increase and decrease over time as patients may enter and leave these states. (A) Transition probabilities for patients allocated to the control arm. (B) Transition probabilities for patients allocated to the CLO arm. Ind&Cons, Induction & Consolidation treatment; MRD‐negativity, MRD‐negativity at consolidation; R/R, relapsed/refractory disease; Relapse mortality, all mortality taking place after relapsed/refractory disease.

**FIGURE 6:**
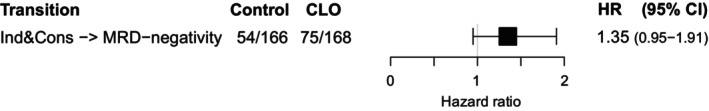
Forest plot for the transition to the MRD‐negativity state as depicted in Figure 2. The estimate shown is a hazard ratio of a semi‐parametric Cox model including treatment arm (CLO vs. control). A hazard ratio less than 1 (left of the vertical line) indicates a lower risk of MRD‐negativity for the CLO arm. The number of patients (relative to number entering the respective state) making the transition to the MRD‐negativity state by treatment arm are tabulated in the middle columns. CI, confidence interval; CLO, number of patients of the CLO arm making the transition; Control, number of patients of the control arm making the transition; HR, hazard ratio.

As shown in Figure 2, all 334 patients started in the Ind&Cons state, whereas 84 patients developed an early relapse or refractory disease during Ind&Cons treatment. In total, 129 patients became MRD‐negative after Ind&Cons and transitioned to the “MRD‐negativity” state of which *n* = 75 (58%) received CLO versus *n* = 54 (42%) received control treatment. As shown in Figure 5, patients in the CLO arm had a somewhat higher probability of becoming MRD‐negative than control patients, and a larger proportion of CLO patients remained MRD‐negative over time as compared to patients in the control arm. The hazard ratio (CLO vs. control) was estimated for the transition from Ind&Cons to the MRD‐negativity state (Figure 6). Patients who were treated with CLO showed a trend towards more MRD‐negativity during consolidation chemotherapy (HR: 1.35 [0.95–1.91], *p* = 0.10). A sensitivity analysis in patients with known MRD outcome after consolidation treatment (MRD‐negative: *n* = 129; MRD‐positive: *n* = 28) did not alter this result. HRs comparing CLO versus control treatment for all transitions are shown in Table S5.

The effect of MRD status on the transition to relapsed/refractory disease was investigated by evaluating the transitions from Ind&Cons and the MRD‐negativity state to relapsed/refractory disease. MRD status in general (i.e., being assessed as either MRD‐positive or MRD‐missing) after consolidation was associated with a 1.6‐fold increased risk of relapsed/refractory disease (HR: 1.63 [1.02–2.63], *p* = 0.042), relative to MRD‐negative patients. As shown in Figure 2, this effect of MRD status was observed by comparing 84 out of 334 (25%) patients (assessed as MRD‐positive [*n* = 14] or MRD‐missing [*n* = 70]) who developed relapsed/refractory disease after Ind&Cons versus 26 out of 129 (20%) patients who developed relapsed/refractory disease after obtaining MRD‐negativity. The trend towards more MRD‐negativity in patients receiving CLO did not translate into better EFS. Lastly, CLO appeared associated with a non‐significant reduced risk of relapsed/refractory disease and RM as compared to patients in the control treatment arm (28% vs. 35% at 4 years, respectively) (Figure 5A vs. 5B, Table S4).

## 4 DISCUSSION

Two cycles of CLO added to standard prephase/induction and consolidation chemotherapy in the randomized HOVON‐100 trial for adult patients with newly diagnosed ALL did not improve EFS or OS compared with control treatment.^1^ While CLO was associated with increased MRD‐negativity, no reduction of relapse was observed. Here, we analyzed the HOVON‐100 ALL trial using multi‐state modeling to investigate in depth the effects of CLO on intermediate events and subsequent treatment outcome. We addressed the effect of CLO on going off‐protocol as a proxy of cumulative toxicities (i.e., treatment risk) and achievement of MRD‐negativity (i.e., treatment benefit), relapsed/refractory disease, and the impact of these intermediate events on EFS. We observed that CLO was associated with an overall increased risk of going off‐protocol (HR: 2.00, *p* = 0.02). This risk was non‐significantly increased during Ind&Cons (HR: 1.68, *p* = 0.15), but CLO was associated with an almost three‐fold higher risk of going off‐protocol during the maintenance phase (HR: 2.85, *p* = 0.035). Going off‐protocol was, however, not associated with relapsed/refractory disease or EFS. We also observed a trend towards more MRD‐negativity by CLO compared with control treatment. While MRD‐negativity in general was associated with less relapse, MRD‐negativity in patients receiving CLO could not be demonstrated to translate into better outcome. Our initial hypothesis that CLO might be associated with increased treatment‐related mortality at the cost of an improved antileukemic effect could not be demonstrated. CLO was associated with an increased risk of treatment‐related toxicity as determined by an increased proportion of patients going off‐protocol. This, however, was not associated with an increase in treatment‐related mortality. Likewise, CLO was associated with an increased rate of MRD‐negativity, but this was not associated with a significant reduction of relapse or improvement of EFS. Our multi‐state analysis thereby shows that, in contrast to our findings in AML,^4^ CLO appeared to exert minor antileukemic effects and no increase in treatment‐related mortality.

Patients randomized to CLO went off‐protocol more frequently than control patients,^1^ which occurred predominantly during maintenance. A higher incidence of infections was observed as adverse advents 30 days prior to going off protocol in the CLO arm. CLO has earlier been associated with Grade 4–5 infections,^8^ and hepatic toxicity without increasing anti‐leukemic efficacy.^9^ The relatively late timing of going off‐protocol most likely reflects cumulative treatment toxicities, which may be a combined effect by CLO and repeated administrations of high‐dose methotrexate and PEG‐asparaginase, on top of which the addition of CLO might be too toxic. Surprisingly, going off‐protocol did not seem to be associated with an increased risk of relapsed/refractory disease, or a reduction of EFS. We hypothesize that this might be due to rescue by alloSCT,^10^, ^11^, ^12^ which was performed in 42% of patients that went off‐protocol.

The initial analysis of the HOVON‐100 trial showed a statistically significantly increased rate of MRD‐negativity by CLO when analyzed selectively in patients who achieved a CR (*p* = 0.01).^1^ In our study, the association between CLO and MRD‐negativity, based on the transition from Ind&Cons to the MRD‐negativity state, was not significant (HR: 1.35, *p* = 0.10). However in our multi‐state analysis, we did not selectively focus on CR patients and considered patients with missing MRD status as MRD‐positive and compared those patients with MRD‐negative patients, consistent with an intention‐to‐treat approach. Comparing MRD‐negative patients with a combination of patients with MRD‐positive and MRD‐missing response after consolidation treatment might have diluted the effect of MRD‐negativity. The impact of CLO on MRD negativity may be explained by its previously well‐documented anti‐leukemic effects.^5^, ^13^, ^14^, ^15^ In this study, MRD‐negativity, irrespective of treatment arm, was significantly associated with reduced relapsed/refractory disease, but not with improved EFS. This may be explained by the restricted availability of MRD‐data in 47% of patients, but also by the intensity of a pediatric‐inspired chemotherapy that was administered for a prolonged period, which allowed patients to benefit also at later time points. In addition, the relatively high number of patients proceeding to alloSCT may have reduced relapse in both early and late MRD responders. Although these data are in contrast with previous observations of MRD as an important prognostic tool or marker for risk stratification and treatment allocation in ALL,^16^, ^17^, ^18^, ^19^, ^20^ our observations with CLO align with a recent multicenter trial of CLO in newly diagnosed pediatric ALL patients that showed a higher MRD negativity rate in patients treated with CLO but without an impact on EFS or OS.^21^

Intensified pediatric‐inspired chemotherapy has considerably improved outcome in adult ALL,^22^ but prevention of relapse has remained an important challenge. Development and evaluation of new drugs in the context of intensive chemotherapy may focus on eradication of MRD and evaluation in MRD‐positive patients (e.g., blinatumomab^23^, ^24^), or on adaptation of drug combinations that may be associated with reduced toxicity. Regarding CLO, the cumulative toxicity with other drugs such as asparaginase and methotrexate, should be avoided. As these drugs are cornerstones of current ALL treatment, it might imply that CLO might be further investigated either in MRD‐positive patients or in the relapse setting or as part of maintenance chemotherapy with less intensive chemotherapy.

Multi‐state modeling may have several advantages over traditional statistical analyses, such as Cox models and landmark analyses.^4^, ^25^, ^26^, ^27^, ^28^ The main advantage is that multi‐state modeling allows for modeling (baseline) hazards separately, and can translate the occurrence of intermediate events and endpoints into probabilities, compared with relative risks by Cox regression. Another advantage of multi‐state modeling is that composite endpoints, such as current leukemia‐free survival,^4^, ^29^ or GvHD‐free, relapse‐free survival,^30^ may be easily derived, by incorporating corresponding states into the multi‐state model. This requires clinical data that incorporate both the occurrence and the exact timing of (intermediate) events. However, multi‐state modeling may suffer from intermediate states and transitions with relatively small patient numbers, even if the overall trial population was reasonably large. A relatively small sample size may also hamper the inclusion of covariates of interest other than treatment arm in a Cox model, including in this study variables such as age, ALL risk classification, or cell of origin, which are all relevant prognostic factors associated with distinct outcome.

In conclusion, a multi‐state analysis newly identified that CLO was associated with increased cumulative toxicities as determined by an increased proportion of patients going to off‐protocol treatment not due to relapsed/refractory disease, especially during the maintenance phase. Despite a trend towards more MRD‐negativity in patients treated with CLO, no improvement of survival outcome estimates was obtained. We concluded that the intermediate states, i.e., going off‐protocol and MRD‐negativity, were affected by adding CLO, but these transitions did not firmly associate with significant adverse or beneficial effects on subsequent survival estimates.

## AUTHOR CONTRIBUTIONS

**Sjoerd J. F. Hermans:** Conceptualization (equal); formal analysis (lead); visualization (lead); writing – original draft (lead). **Yvette van Norden:** Conceptualization (equal); formal analysis (supporting); supervision (lead); writing – review and editing (equal). **Jurjen Versluis:** Supervision (lead); writing – review and editing (equal). **Anita W. Rijneveld:** Writing – review and editing (equal). **Bronno van der Holt:** Supervision (supporting); writing – review and editing (equal). **Okke de Weerdt:** Writing – review and editing (equal). **Bart J. Biemond:** Writing – review and editing (equal). **Arjan A. van de Loosdrecht:** Writing – review and editing (equal). **Lotte E. van der Wagen:** Writing – review and editing (equal). **Mar Bellido:** Writing – review and editing (equal). **Michel van Gelder:** Writing – review and editing (equal). **Walter J. F. M. van der Velden:** Writing – review and editing (equal). **Dominik Selleslag:** Writing – review and editing (equal). **Daniëlle van Lammeren‐Venema:** Writing – review and editing (equal). **Vincent H. J. van der Velden:** Writing – review and editing (equal). **Liesbeth C. de Wreede:** Supervision (supporting); writing – review and editing (equal). **Douwe Postmus:** Supervision (supporting); writing – review and editing (equal). **Francesco Pignatti:** Writing – review and editing (equal). **Jan J. Cornelissen:** Supervision (lead); writing – review and editing (equal).

## CONFLICT OF INTEREST STATEMENT

All authors have no competing financial interests to declare related to this work.

## ETHICS STATEMENT

The novel research presented is fully covered by the original ethical approval of the HOVON 100 RCT, as documented in the paper by Rijneveld et al., Blood Adv., 2022: “*The study protocol was approved by independent ethics committees at each participating center, and the study was conducted in accordance with the Declaration of Helsinki. All participants provided written informed consent*.”

## TRIAL REGISTRATION

The HOVON 100 RCT was registered at www.trialregister.nl as #NTR2004.

## Supporting information


Data S1.

## Data Availability

The data that support the findings of this study are available on request from the corresponding author. The data are not publicly available due to privacy or ethical restrictions.
