# Supplementary material for: Benefits and risks of clofarabine in adult acute lymphoblastic leukemia investigated in depth by multi‐state modeling
Source: Cancer Med. 2024 Apr 29;13(9):e6756. doi: 10.1002/cam4.6756 (PMC11056700; doi:10.1002/cam4.6756)
Supplement: Supplementary file 1 — Data S1. [file CAM4-13-e6756-s001.pdf]

Supplementary Appendix

This is the appendix to Hermans et al. - Benefits and risks of clofarabine in adult ALL investigated in depth by multi-state modeling

Table of contents

Supplementary Table 1. Entry criteria per state for the "off-protocol treatment" and "MRD" multi-state models 3

Supplementary Table 2. Transition probabilities and 95% confidence intervals at 2 and 4 years for patients starting in induction/consolidation chemotherapy at time 0 for the multi-state off-protocol treatment model 4

Supplementary Table 3. Hazard ratios and 95% confidence intervals for all transitions corresponding to the multi-state off-protocol treatment model 5

Supplementary Table 4. Transition probabilities and 95% confidence intervals at 2 and 4 years for patients starting in induction/consolidation chemotherapy at time 0 for the multi-state MRD model 6

Supplementary Table 5. Hazard ratios and 95% confidence intervals for all transitions corresponding to the multi-state MRD model 7

Supplementary Figure 1. Extended multi-state model for off-protocol treatment including alloSCT and maintenance treatment 8

Supplementary Figure 2. Transition probabilities to all states from Ind&Cons relating to the extended “off-protocol treatment” model (as depicted in Supplementary Figure 1) 9

Supplementary Figure 3. Forest plot for the transitions to the off-protocol treatment state (as depicted in Supplementary Figure 1) 10

Supplementary Table 6. Entry criteria per state for the "off-protocol treatment" multi-state model including alloSCT and maintenance treatment 11

Supplementary Table 7. Transition probabilities and 95% confidence intervals at 2 and 4 years for patients starting in induction/consolidation chemotherapy at time 0 for the multi-state off-protocol treatment model including alloSCT and maintenance treatment 12

Supplementary Table 8. Hazard ratios and 95% confidence intervals for all transitions corresponding to the multi-state off-protocol treatment model including alloSCT and maintenance treatment 13

Supplementary Table 1. Entry criteria per state for the "off-protocol treatment" and "MRD" multi-state models

State	Off-protocol treatment model	MRD model
Ind&Cons	Having started on-protocol induction & consolidation chemotherapy	Having started on-protocol induction & consolidation chemotherapy
Off-protocol treatment	Going off-protocol for reasons other than relapse, death, or on-protocol treatment completion; no refractory disease or on-protocol alloSCT / maintenance chemotherapy	Not applicable
MRD-negativity	Not applicable	Negative MRD assessment after consolidation chemotherapy
Relapsed/refractory disease	Development of relapsed/refractory disease during follow-up	Development of relapsed/refractory disease during follow-up
RM	Any death following relapsed/refractory disease development	Any death following relapsed/refractory disease development
NRM	Any death without prior relapsed/refractory disease development	Any death without prior relapsed/refractory disease development

AlloSCT: allogeneic stem cell transplant; Ind&Cons: induction and consolidation chemotherapy; MRD: minimal residual disease; NRM: non-relapse mortality; RM: Relapse Mortality

Entry criteria for each state of the multi-state "off-protocol treatment" and "MRD" models are tabulated. All patients started in Ind&Cons at time 0 and could enter a subsequent state only following relapsed/refractory ALL, going off-protocol, MRD-negativity, or NRM. Patients who did not qualify for a subsequent state remained in the preceding state.

Supplementary Table 2. Transition probabilities and 95% confidence intervals at 2 and 4 years for patients starting in induction/consolidation chemotherapy at time 0 for the multi-state off-protocol treatment model

	**2 years**		**4 years**	
**Transition**	Control	CLO	Control	CLO
Ind&Cons	54.6 (47.9-62.2)	50.2 (43.6-57.9)	45.4 (38.5-53.4)	41.4 (34.7-49.3)
Relapsed/refractory disease	9.6 (6.1-15.3)	8.6 (5.3-14.1)	10.2 (6.3-16.7)	9.2 (5.5-15.7)
RM	17.9 (13.3-24.2)	13.7 (9.7-19.3)	24.7 (19.1-31.9)	19.3 (14.3-26.0)
Off-protocol treatment	5.0 (2.6-9.5)	11.7 (7.7-17.7)	5.1 (2.5-10.4)	12.3 (8.0-18.9)
NRM	12.9 (9.0-18.6)	15.8 (11.3-22.0)	14.6 (10.3-20.7)	17.8 (13.0-24.5)

Ind&Cons: induction and consolidation chemotherapy; NRM: non-relapse mortality; RM: relapse mortality

Transition probabilities and 95% confidence intervals at 2 and 4 years for patients starting in induction/consolidation chemotherapy at time 0 for the multi-state off-protocol treatment model are tabulated. These transition probabilities are the numerical representation of the transition probabilities shown in Figure 3.

Supplementary Table 3. Hazard ratios and 95% confidence intervals for all transitions corresponding to the multi-state off-protocol treatment model

**Transition**	HR	95% CI	P
Ind&Cons → Relapsed/refractory disease	0.76	0.51-1.14	0.18
Ind&Cons → Off-protocol treatment	2.00	1.13-3.52	0.02
Ind&Cons → NRM	1.28	0.72-2.27	0.40
Relapsed/refractory disease → RM	0.93	0.59-1.46	0.75
Off-protocol treatment → Relapsed/refractory disease	0.79	0.26-2.42	0.68
Off-protocol treatment → NRM	0.53	0.15-1.84	0.32

Ind&Cons: induction and consolidation chemotherapy; NRM: non-relapse mortality;

RM: relapse mortality

Hazard ratios and 95% confidence intervals for all transitions corresponding to the multi-state off-protocol treatment model (Figure 1) are tabulated. The estimates were calculated using a transition-specific Cox model including treatment arm (CLO vs. control). A hazard ratio smaller than 1 indicates a lower hazard ratio for the CLO arm.

Supplementary Table 4. Transition probabilities and 95% confidence intervals at 2 and 4 years for patients starting in induction/consolidation chemotherapy at time 0 for the multi-state MRD model

	**2 years**		**4 years**	
**Transition**	Control	CLO	Control	CLO
Ind&Cons	34.2 (28.0-41.9)	29.8 (23.9-37.1)	27.6 (21.8-35.0)	25.0 (19.4-32.2)
Relapsed/refractory disease	9.8 (6.2-15.3)	8.4 (5.2-13.6)	10.2 (6.4-16.5)	9.0 (5.3-15.1)
RM	18.0 (13.4-24.2)	13.6 (9.7-19.0)	24.8 (19.2-31.9)	19.0 (14.2-25.6)
MRD-negativity at consolidation	24.9 (19.2-32.2)	32.4 (26.4-39.8)	22.5 (17.0-29.7)	29.3 (23.4-36.7)
NRM	13.1 (9.2-18.8)	15.8 (11.4-21.7)	14.9 (10.6-20.9)	17.7 (13.0-24.1)

Ind&Cons: induction and consolidation chemotherapy; MRD: minimal residual disease; NRM: non-relapse mortality; RM: relapse mortality

Transition probabilities and 95% confidence intervals at 2 and 4 years for patients starting in induction/consolidation chemotherapy at time 0 for the multi-state MRD model are tabulated. These transition probabilities are the numerical representation of the transition probabilities shown in Figure 5.

Supplementary Table 5. Hazard ratios and 95% confidence intervals for all transitions corresponding to the multi-state MRD model

**Transition**	HR	95% CI	P
Ind&Cons → Relapsed/refractory disease	0.73	0.47-1.13	0.15
Ind&Cons → MRD-negativity at consolidation	1.35	0.95-1.91	0.10
Ind&Cons → NRM	1.50	0.78-2.88	0.22
Relapsed/refractory disease → RM	0.93	0.59-1.46	0.75
MRD-negativity at consolidation → Relapsed/refractory disease	1.19	0.54-2.63	0.67
MRD-negativity at consolidation → NRM	0.73	0.30-1.75	0.48

Ind&Cons: induction and consolidation chemotherapy; MRD: minimal residual disease; NRM: non-relapse mortality; RM: relapse mortality

Hazard ratios and 95% confidence intervals for all transitions corresponding to the multi-state MRD model (Figure 2) are tabulated. The estimates were calculated using a transition-specific Cox model including treatment arm (CLO vs. control). A hazard ratio smaller than 1 indicates a lower hazard ratio for the CLO arm.

Supplementary Figure 1. Extended multi-state model for off-protocol treatment including alloSCT and maintenance treatment

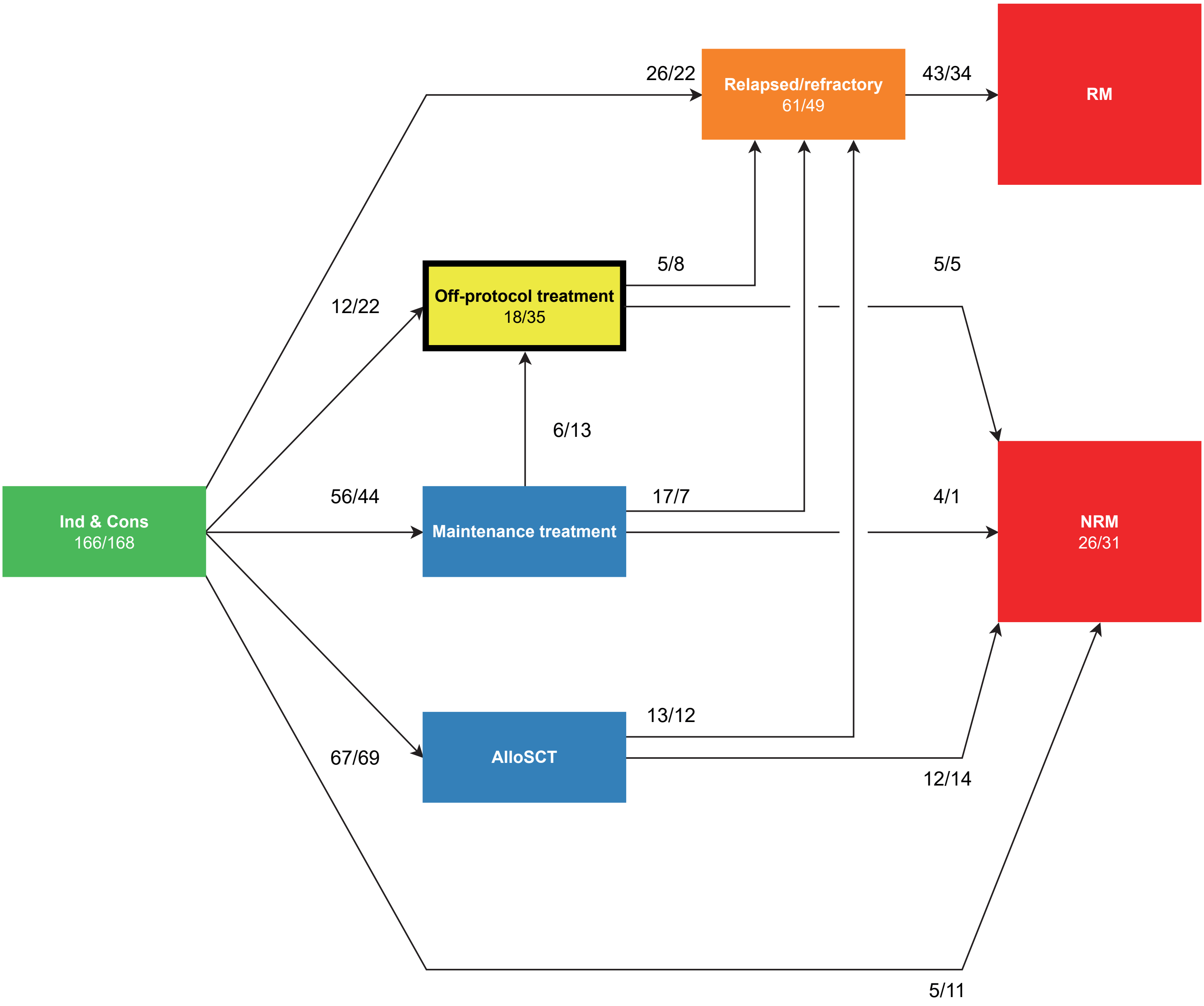

A time-inhomogeneous Markov model for the events between Induction & Consolidation (Ind&Cons) and date of last contact was developed. All patients started in Ind&Cons at time 0 and could enter a subsequent state only following relapsed/refractory ALL, going off-protocol, start of maintenance treatment, alloSCT, or NRM. Patients who did not qualify for a subsequent state remained in the preceding state. Going off-protocol for reasons other than relapsed/refractory disease, death or treatment completion was used as a proxy of treatment-related toxicity by CLO. Going off-protocol, maintenance treatment, alloSCT and relapsed/refractory ALL were used as intermediate events, while RM and NRM were modeled as end states. Event counts per treatment arm ("control vs. CLO") are listed for each transition. AlloSCT: allogeneic stem cell transplant; NRM: non-relapse mortality; RM: relapse mortality (all mortality taking place after relapsed/refractory disease)

Supplementary Figure 2. Transition probabilities to all states from Ind&Cons relating to the extended "off-protocol treatment" model (as depicted in Supplementary Figure 1)

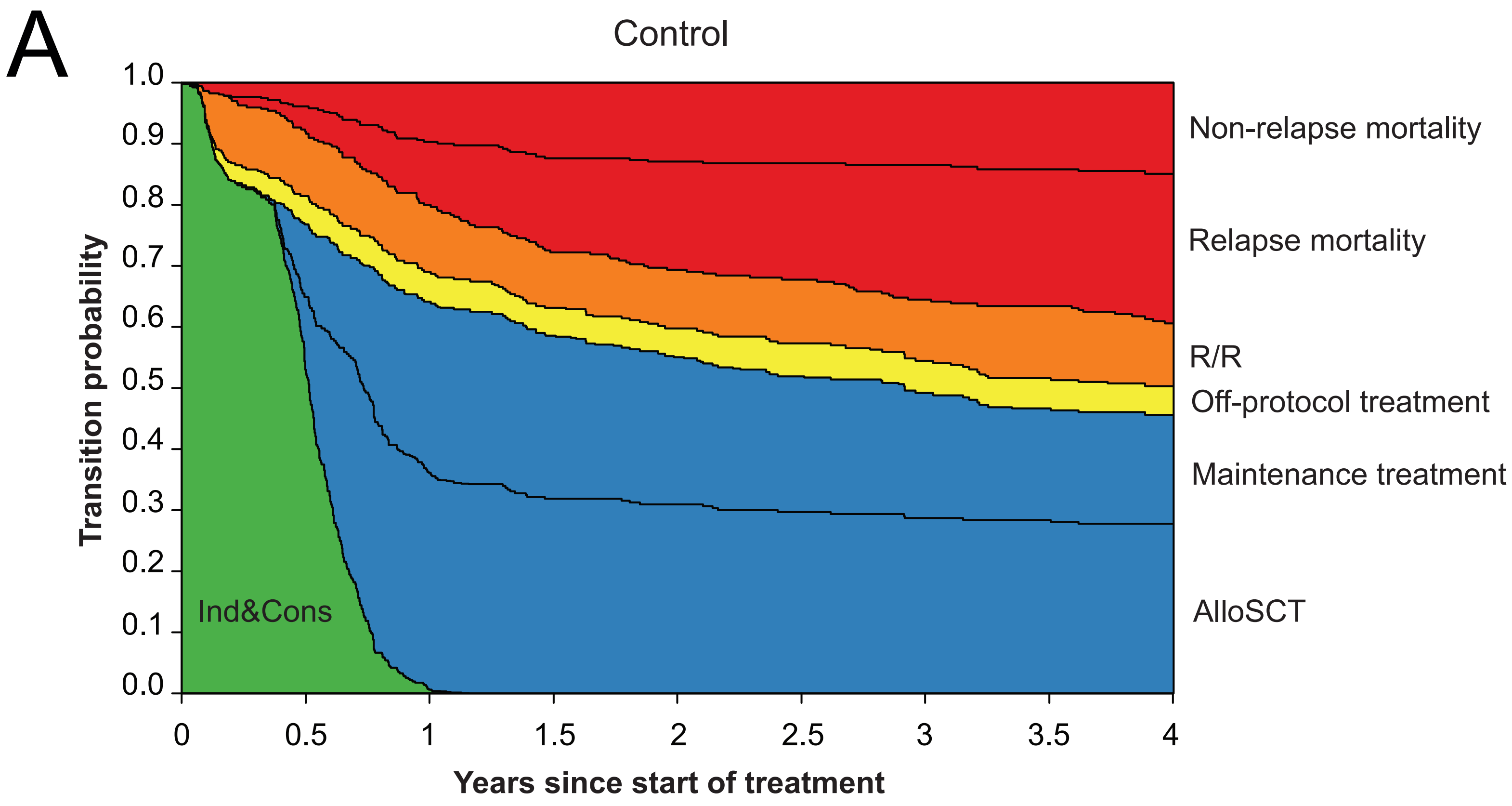

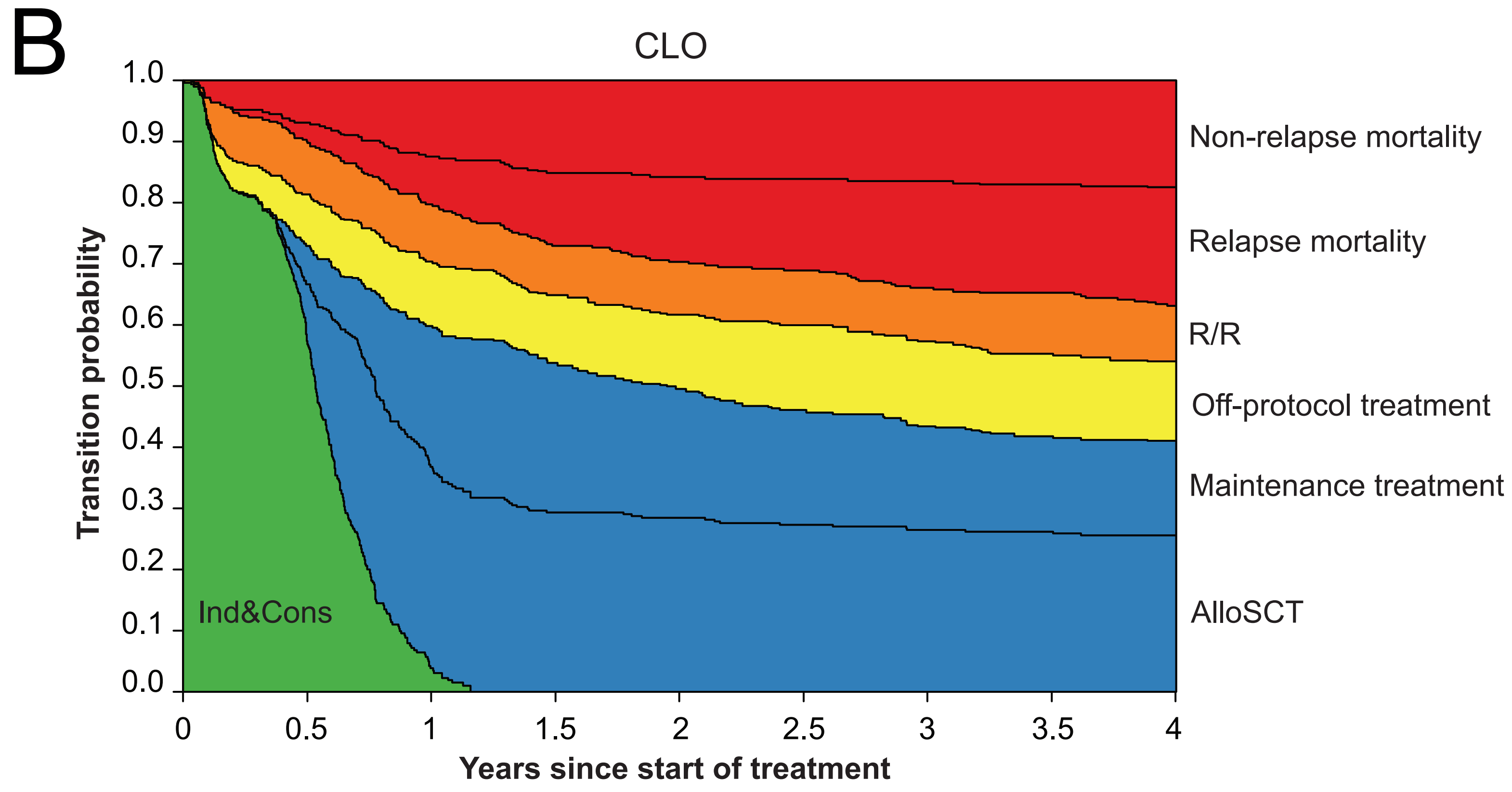

Starting in Ind&Cons at time 0, the distance between neighboring lines depicts the probability of being in that state, at each time point. Probabilities of intermediate states can both increase and decrease over time as patients may enter and leave these states. Panel A: transition probabilities for patients allocated to the control arm. Panel B: transition probabilities for patients allocated to the CLO arm. AlloSCT: allogeneic stem cell transplant; Ind&Cons: Induction & Consolidation treatment; R/R: relapsed/refractory disease; Relapse mortality: all mortality taking place after relapsed/refractory disease

Supplementary Figure 3. Forest plot for the transitions to the off-protocol treatment state (as depicted in Supplementary Figure 1)

Transition	Control	CLO	HR (95% CI)
Ind&Cons −> Off−protocol	12/166	22/168	1.68 (0.83-3.40)
Maintenance −> Off−protocol	6/56	13/44	2.85 (1.08−7.50)*

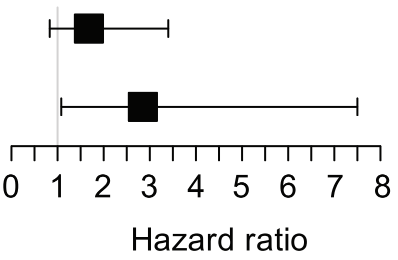

Estimates shown are hazard ratios of a semi-parametric Cox model including treatment arm (CLO vs. control). A hazard ratio smaller than 1 (left of the vertical line) indicates a lower risk of going off-protocol for the CLO arm. The number of patients (relative to number entering the respective state) making the transition to the off-protocol state by treatment arm are tabulated in the middle columns. *: the transition from maintenance treatment to the off-protocol treatment state was the only transition with a P-value less than 0.05 (0.035). Control: number of patients of the control arm making the transition; CLO: number of patients of the CLO arm making the transition; CI: confidence interval; HR: hazard ratio; Ind&Cons: induction & consolidation treatment

Supplementary Table 6. Entry criteria per state for the "off-protocol treatment" multi-state model including alloSCT and maintenance treatment

State	Off-protocol treatment model
Ind&Cons	Having started on-protocol induction & consolidation chemotherapy
Off-protocol treatment	Going off-protocol for reasons other than relapse, death, or on-protocol treatment completion; no refractory disease or on-protocol alloSCT / maintenance chemotherapy
Maintenance treatment	Having started on-protocol maintenance treatment before alloSCT and without prior development of relapsed/refractory disease
AlloSCT	Having started on-protocol alloSCT before maintenance treatment and without prior development of relapsed/refractory disease
Relapsed/refractory disease	Development of relapsed/refractory disease during follow-up
RM	Any death following relapsed/refractory disease development
NRM	Any death without prior relapsed/refractory disease development

AlloSCT: allogeneic stem cell transplant; Ind&Cons: induction and consolidation chemotherapy; MRD: minimal residual disease; NRM: non-relapse mortality; RM: Relapse Mortality

Entry criteria for each state of the extended multi-state "off-protocol treatment" model are tabulated. All patients started in Ind&Cons at time 0 and could enter a subsequent state only following relapsed/refractory ALL, going off-protocol, maintenance treatment, alloSCT, or NRM. Patients who did not qualify for a subsequent state remained in the preceding state.

Supplementary Table 7. Transition probabilities and 95% confidence intervals at 2 and 4 years for patients starting in induction/consolidation chemotherapy at time 0 for the multi-state off-protocol treatment model including alloSCT and maintenance treatment

	**2 years**		**4 years**	
**Transition**	Control	CLO	Control	CLO
Ind&Cons	0	0	0	0
Relapsed/refractory disease	9.6 (6.1-15.1)	8.6 (5.2-14.3)	10.3 (6.3-16.8)	9.1 (5.2-15.8)
RM	17.7 (13.0-24.2)	13.9 (9.7-19.8)	24.5 (18.8-31.9)	19.4 (14.2-26.5)
Off-protocol treatment	4.7 (2.5-8.9)	12.1 (8.1-18.2)	4.7 (2.3-9.6)	13.0 (8.5-19.8)
Maintenance treatment	24.1 (18.9-30.7)	21.1 (16.1-27.6)	17.8 (13.1-24.2)	15.5 (10.9-21.9)
AlloSCT	31.0 (24.9-38.5)	28.5 (22.4-36.2)	27.8 (21.8-35.5)	25.6 (19.6-33.4)
NRM	12.9 (8.7-19.2)	15.8 (10.9-22.9)	14.9 (10.3-21.6)	17.4 (12.2-25.0)

AlloSCT: allogeneic stem cell transplant; Ind&Cons: induction and consolidation chemotherapy; NRM: non-relapse mortality; RM: relapse mortality

Transition probabilities and 95% confidence intervals at 2 and 4 years for patients starting in induction/consolidation chemotherapy at time 0 for the extended multi-state "off-protocol treatment" model are tabulated. These transition probabilities are the numerical representation of the transition probabilities shown in Supplementary Figure 2.

Supplementary Table 8. Hazard ratios and 95% confidence intervals for all transitions corresponding to the multi-state off-protocol treatment model including alloSCT and maintenance treatment

**Transition**	HR	95% CI	P
Ind&Cons → Relapsed/refractory disease	0.78	0.44-1.37	0.38
Ind&Cons → Off-protocol treatment	1.68	0.83-3.40	0.15
Ind&Cons → Maintenance treatment	0.52	0.35-0.78	0.0017
Ind&Cons → AlloSCT	0.76	0.54-1.06	0.11
Ind&Cons → NRM	2.09	0.73-6.02	0.17
Relapsed/refractory disease → RM	0.93	0.59-1.46	0.75
Off-protocol treatment → Relapsed/refractory disease	0.79	0.26-2.42	0.68
Off-protocol treatment → NRM	0.53	0.15-1.84	0.32
Maintenance treatment → Relapsed/refractory disease	0.56	0.23-1.36	0.20
Maintenance treatment → Off-protocol treatment	2.85	1.08-7.50	0.035
Maintenance treatment → NRM	0.37	0.04-3.29	0.37
AlloSCT → Relapsed/refractory disease	0.94	0.43-2.08	0.88
AlloSCT → NRM	1.20	0.55-2.62	0.64

AlloSCT: allogeneic stem cell transplant; Ind&Cons: induction and consolidation chemotherapy; NRM: non-relapse mortality; RM: relapse mortality

Hazard ratios and 95% confidence intervals for all transitions corresponding to the extended multi-state “off-protocol treatment” model (Supplementary Figure 1) are tabulated. The estimates were calculated using a transition-specific Cox model including treatment arm (CLO vs. control). A hazard ratio smaller than 1 indicates a lower hazard ratio for the CLO arm.
